# Quantitative evaluation of the dark rim artifact in cardiac perfusion images

**DOI:** 10.1186/1532-429X-11-S1-P243

**Published:** 2009-01-28

**Authors:** Raphael D Hazel, Nathaniel Reichek, Yi Wang

**Affiliations:** St Francis Hospital, Roslyn, NY USA

**Keywords:** Signal Loss, Myocardial Perfusion Image, Myocardial Blood Flow, Normal Volunteer, Short Axis Slice

## Introduction

The dark rim artifact (DRA) which appears in cardiac first pass perfusion MRI compromises both visual assessment and quantitative evaluation of ischemia. DRA signal loss in the subendocardium may be mistaken for a perfusion defect or it can overlap with a region showing a real perfusion disorder, and make it impossible to obtain an accurate assessment of myocardial blood flow. The quantitatively evaluation of the artifact will allow for precise determination of its origin, automated image assessment, and elimination of observer error from the evaluation of cardiac perfusion images. We have quantified the DRA in perfusion images from three separate studies using SSFP, TFL and EPI imaging sequences to determine the characteristics and origin of the artifact.

## Methods

Retrospective quantitative analysis was done on myocardial perfusion images from 32 normal volunteers. Images from three separate studies approved by the institutional review board were analyzed for artifacts. In the first study, images were acquired in 14 normal volunteers (10 female) age 43.8 (s.d. 15.9) years, using a saturation recovery SSFP pulse sequence and gadodiamide (0.05 mM/kg), under adenosine (140 ug/kg/min) stress followed by rest. The image parameters were as follows: body coil, TR 160 ms, TE 1.03 ms, FA 50, matrix 192 × 108; three long axis planes (HLA, VLA, and LVOT) of 8 mm slice thickness were recorded. In the second study, using the same imaging parameters, TFI images from 7 subjects which were acquired at rest, under cold pressor stress, and after 0.4 mg sublingual nitroglycerin were analyzed. In the third study, images were acquired using the dual bolus approach with two doses of gadodiamide (0.005 and 0.05 mM/kg) at rest from 11 normal volunteers (5 female) using the TFI, TFL and EPI pulse sequences in random order during a single session. Imaging parameters for each sequence were as follows: TFI (TR 199 ms, TE 1.04 ms, FA 50, matrix 192 × 160) TFL (TR 163 ms, TE 1.27 ms, FA 10, matrix 192 × 160) and EPI (TR 5.8 ms, TE 1.22 ms, FA 25, matrix 160 × 132) Three long axis and one short axis slice with thicknesses of 10 mm were collected. Images were analyzed for the presence of DRA using ImageJ to measure the intensity profile across the myocardium. DRA was identified as signal loss in the subendocardium occurring after the arrival of the CA in the LV with a duration coincident with the passage of the bolus through the LV.

## Results

### Heart motion

The signal loss per image appears to be independent of heart rate within each study. In study #1 the mean signal loss per image is 32.9% and 31.5% for rest and stress respectively, and in study #2 the signal loss per image was 21.9%, 21.3% and 20.8% for rest, cold stress and nitroglycerine respectively.

### Concentration dependence

There were no artifacts in low CA dose (0.005 mM/kg) images. Only the high CA dose produced the DRA in TFI and TFL images.

### Sequence dependence

DRA was found in TFI images from 8 of 11 volunteers (4% of all images) while only 3 sets of TFL (2%) images contained the DRA. EPI images did not contain the DRA. The severity of the DRA is similar for TFI and TFL images with a mean signal loss of 31.6% and 33.1% respectively. The average duration of the DRA is also similar for TFI and TFL with 11.8 and 10.5 frames out of 50 respectively.

## Conclusion

The signal loss in the DRA is independent of heart rate, but seems to depend on CA concentration for TFI and TFL but not EPI images. Quantitative evaluation of the DRA is feasible with first pass perfusion imaging.

